# Function and return to sports after proximal humeral replacement in patients with primary bone sarcoma

**DOI:** 10.1186/s10195-022-00678-z

**Published:** 2022-12-26

**Authors:** Moritz Ellerbrock, Christoph Theil, Georg Gosheger, Niklas Deventer, Sebastian Klingebiel, Carolin Rickert, Kristian Nikolaus Schneider

**Affiliations:** Department of Orthopaedics and Tumor Orthopaedics, Albert-Schweitzer Campus 1, 48149 Münster, Germany

**Keywords:** Megaprosthesis, Humerus, Bone sarcoma, Return to sport, Functional outcome

## Abstract

**Background:**

Improved patient and limb survival rates have led to an increased interest in the functional outcome and return to sports of patients undergoing megaprosthetic reconstruction in musculoskeletal oncology. This study evaluates the functional outcome and postoperatively performed level of sports in patients undergoing proximal humeral replacement (PHR) following resection of a primary bone sarcoma and identifies potential beneficial and limiting factors.

**Patients and methods:**

Between 2007 and 2020, a total of 606 patients underwent resection of a primary bone sarcoma and reconstruction with a single-design modular implant. For 112 (18%) patients, the location of the tumour was the proximal humerus. Exclusion criteria were death (*n* = 65), patients living overseas (*n* = 8), and subsequent amputation (*n* = 1), leaving 38 patients for evaluation, of whom 32 were available for the study (13 women, median age 42 years). Clinical data regarding oncological and surgical treatment as well as subsequent complications were obtained from the patients’ electronic medical records. Functional outcome was determined using the Musculoskeletal Tumor Society Score (MSTS) and Toronto Extremity Salvage Score (TESS) as well as the Subjective Shoulder Value (SSV). Return to sports was assessed using the Tegner Activity Score (TS) and the modified Weighted Activity Score (WAS).

**Results:**

At the last follow-up after a median of 30 months (IQR 22–58), median MSTS was 18 (IQR 12–24), median TESS was 80% (IQR 69–87), median SSV was 35% (IQR 10–58), median TS was 5 (IQR 4–6) and median WAS was 5 (IQR 0–10). Preservation of the axillary nerve, a reverse shoulder reconstruction and a WAS of > 10 prior to surgery were associated with better functional outcome and return to sports activity scores.

**Conclusion:**

Following PHR, good to excellent functional outcomes are possible, and patients regularly return to participate in sports activities—most commonly in low-impact types of sports, but some individuals are even able to participate in high-impact sports activities.

**Level of evidence:**

IV.

**Supplementary Information:**

The online version contains supplementary material available at 10.1186/s10195-022-00678-z.

## Introduction

The humerus is the third most common site of primary malignant bone tumours [1, 2]. Treatment of these entities is usually defined by multimodal study protocols, and surgical treatment commonly requires excision of the tumour with wide surgical margins [3]. A common mode of reconstruction is to use a modular, megaprosthetic replacement with a proximal humerus replacement [4, 5]. Over the past decades, advances in oncological and surgical treatment have resulted in improved limb and patient survival rates [6]*.* These achievements have subsequently led to an increased interest in patients’ postoperative function and return to sports activities and the potential factors associated with a successful return to sports activities—especially considering that mostly young patients are affected by primary bone sarcomas [7–10]. However, while sports activities in sarcoma patients who have undergone megaprosthetic reconstruction have previously been studied for lower extremity tumours, there is a paucity of studies on upper extremity reconstructions, particularly studies applying homogeneous inclusion criteria and larger patient cohorts [11, 12]*.* Furthermore, patient- and procedure-related factors associated with outcome are widely unknown. Thus, the aims of the present study were (1) to determine the functional outcome and (2) to evaluate the postoperatively achieved level of sports using standardized scoring systems in a large cohort of patients who had undergone proximal humeral replacement (PHR) following resection of a primary bone sarcoma, and (3) to identify the associated beneficial and limiting factors.

## Patients and methods

**Fig. 1:**
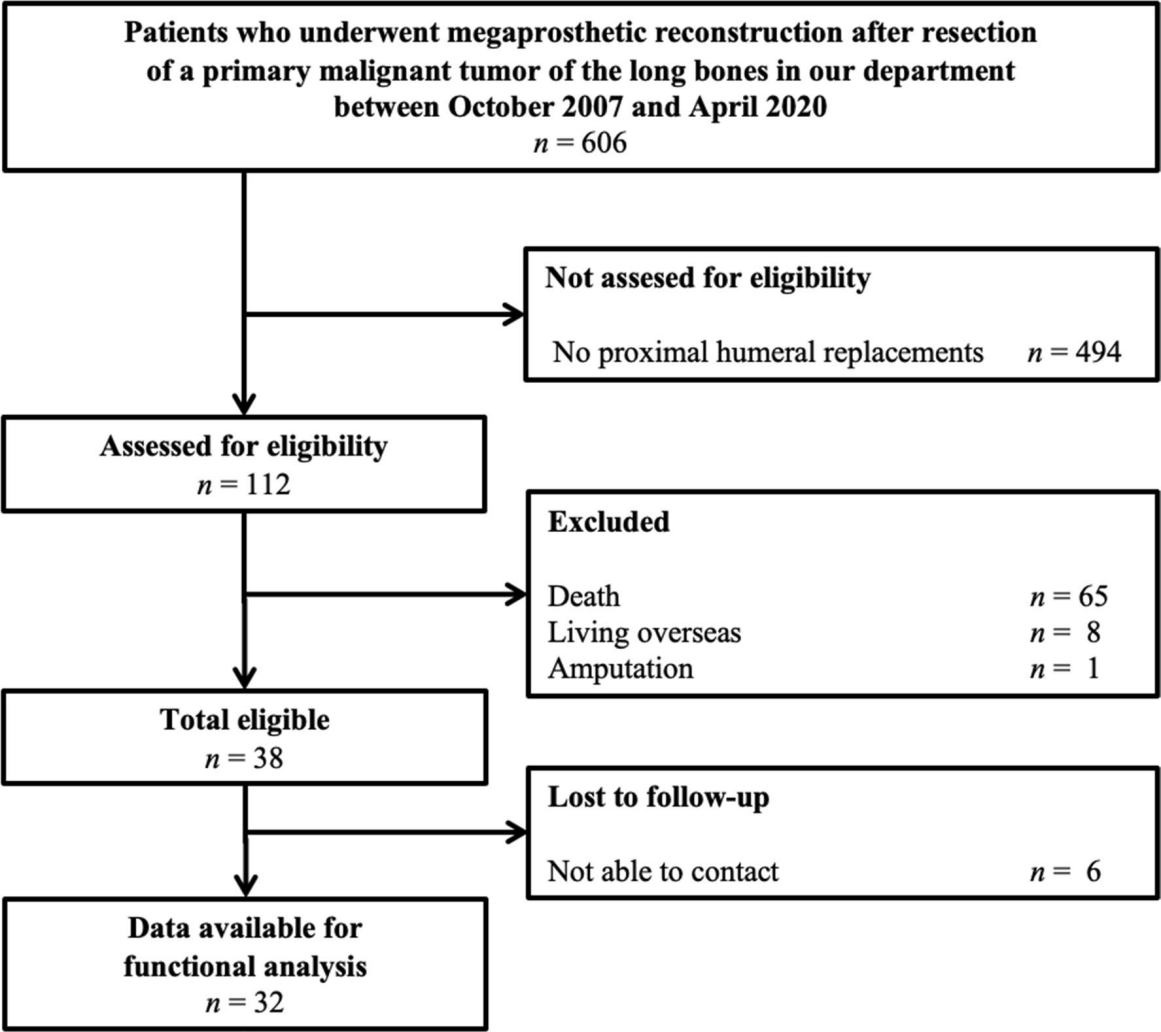
STROBE study flow diagram

Between October 2007 and April 2020, a total of 606 patients underwent resection of a primary bone sarcoma of the long bones and subsequent reconstruction with a single-design modular universal tumour and revision system (MUTARS, Implantcast, Buxtehude, Germany) in our department in a tertiary university hospital. For 112 (18%) of these patients, the location of the tumour was the proximal humerus. Exclusion criteria for the present study were death (*n* = 65), patients living overseas (*n* = 8), and subsequent amputation (*n  *= 1), leaving 38 patients. Six patients could not be contacted for the study, leaving a final cohort of 32 patients (Fig. 1, Table 1).

**Table 1:** Demographics and oncological and surgical details

Variable	*n* (%)
Gender	
Female	13 (41)
Male	19 (59)
Tumour entity	
Osteosarcoma	13 (41)
Chondrosarcoma	10 (31)
Ewing sarcoma	6 (19)
Pleomorphic sarcoma of the bone	1 (3)
Myxofibrosarcoma	1 (3)
Fibromyxosarcoma	1 (3)
Type of tumour resection	
Intra-articular	22 (69)
Extra-articular	10 (31)
Type of shoulder reconstruction	
Anatomic	20 (62)
Reverse	12 (38)

### Surgical technique

All patients underwent a planned wide tumour resection with histopathological confirmation of surgical margins. In order to achieve these wide surgical margins, the axillary nerve had to be resected or could be preserved. In patients with tumour infiltration of the glenohumeral joint, an extraarticular tumour resection was performed. For soft-tissue reconstruction, an attachment tube (Trevira, Implantcast, Buxtehude, Germany) was used [13, 14]. As of 2006, silver-coated implants were available and used in all patients [15]. As of 2010, implants for reverse shoulder reconstruction were available and used in patients in whom the axillary nerve and a sufficient portion of the deltoid could be preserved [11–13].

### Data collection

Clinical data regarding the oncological and surgical treatment as well as subsequent complications were obtained from the patients’ electronic medical records. Functional outcome and pre- and post-operatively performed levels of sports were evaluated using standardized scoring systems.

### Assessment of functional outcome and classification of complications

The functional outcome was determined using the Musculoskeletal Tumor Society Score (MSTS), the Toronto Extremity Salvage Score (TESS) and the Subjective Shoulder Value (SSV). MSTS and TESS are commonly used in tumour orthopaedics to assess the functional outcome following limb-sparing surgery, and there are specifically designed versions of each for upper and lower extremities, respectively [16, 17]*.* The upper extremity version of the MSTS includes six questions on dexterity, pain, emotional acceptance, function, hand positioning and the ability to lift objects with the affected arm [17]. Each question is scored on a scale from 0 (very limited) to 5 (no restriction), with a maximum score of 30 points [17]. The upper extremity version of the TESS includes 29 questions, each scored on a scale from 1 (impossible to do) to 5 (not at all difficult), on everyday upper extremity tasks such as cutting vegetables or doing household chores [16]. The maximum score is 145 points, which is converted to a percentage to allow easier comparability [16]. Furthermore, we obtained the SSV, where a patient rates the function of the operated shoulder (as a percentage) compared to the contralateral shoulder [18].

The performed level of sports activities was assessed using the Tegner Activity Score (TS) and the modified Weighted Activity Score (WAS). For the TS, the patient states his highest level of performed sports on a scale from 1 (cannot move) to 10 (participates in competitive contact sports—national elite level) [19, 20]. The WAS is an individual performance score covering the frequency, duration and type of performed sports (low, medium or high impact) [21]. The score is obtained by multiplying the frequency (per week), duration (in hours) and weighted points based on the respective impact of the performed sports activity—e.g. a sports activity with a low load (e.g. swimming) equals a factor of 1 and a sports activity with a high load (e.g. soccer) equals a factor of 3 (Table 2) [21–23]. If more than one activity was performed, the respective scores for each individual activity were added to the final WAS [21, 22]. WASs from 0 to 10 indicate low-activity patients and WASs higher than 10 indicate high-activity patients.

**Table 2:** Impact of performed sports activities according to Healy et al. [21]

Impact	Type of sports activity
Low	Nordic walking, dancing, diving, aqua gymnastic, swimming, hiking, cycling, fishing, bowling, sailing
Medium	Skiing, canoeing, badminton, golf
High	American football, soccer, tennis, triathlon, judo, basketball, jogging

Endoprosthetic complications were classified according to Henderson et al. as soft tissue failure (type 1), aseptic loosening (type 2), structural failure (type 3), infection (type 4) and tumour progression (type 5) [24]*.*

### Statistical analysis

Statistical analyses were performed using SPSS 25.0 (IBM Corp., Armonk, NY, USA). The duration of follow-up was calculated from the date of surgery to the date of the event or the last documented contact with the patient as of December 2021. The data distribution was determined with the Kolmogorov–Smirnov test. Non-parametric analyses were performed with the Mann–Whitney *U* test, and parametric analyses were performed with Student’s *t* test. All *p* values were two-sided and a *p* value of less than 0.05 was considered statistically significant.

## Results

Results from a total of 32 patients (84% follow-up rate, 13 women) with a median age of 42 years (IQR 24–56) on the day of surgery and a median BMI of 25 (IQR 22–27) were available for analysis after a median follow-up of 30 months (IQR 22–58).

### Functional outcome

The median MSTS before diagnosis was 30 (IQR 28–30) and 18 (IQR 12–24) at final follow-up. The median TESS before diagnosis was 100% (145/145, IQR 100–100) and 80% (116/145, IQR 69–87) at final follow-up. The median SSV at final follow-up was 35% (IQR 10–58).

### Return to sports activities

The median TS prior to diagnosis was 6 (IQR 6–7) and 5 (IQR 4–6) at final follow-up. The median WAS before diagnosis was 10 (IQR 5–21) and 5 (IQR 0–10) at final follow-up.

Prior to surgery, 91% (29/32) of the patients reported that they were active in sports activities. Participants engaged particularly frequently in fitness/gymnastics (16%, 5/32), cycling (13%, 4/32) and swimming (13%, 4/32). Three patients preoperatively performed sports at the competitive tournament level (defined as TS > 8), namely dancing, soccer and American football, respectively.

**Fig. 2:**
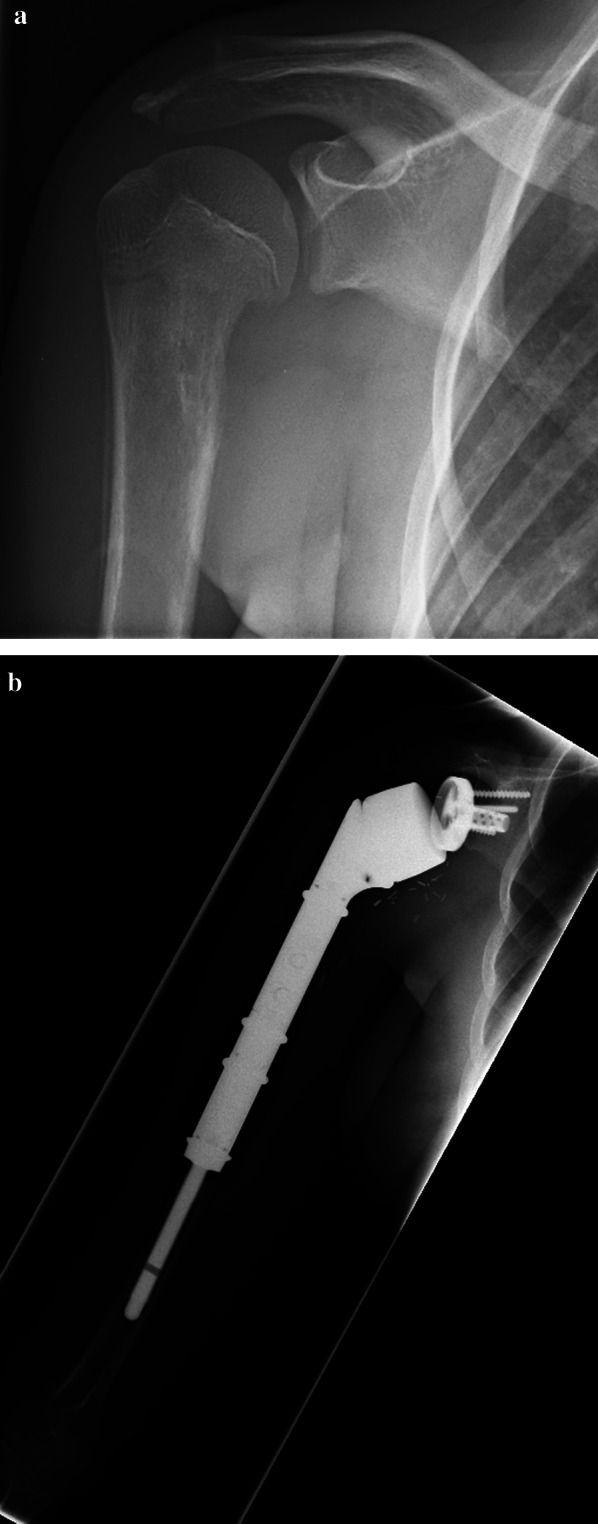
**a** Osteosarcoma of the right proximal humerus in an 18-year-old female patient; **b** radiographic imaging 40 months following PHR

At the time of the last follow-up, 69% (22/32) of the patients participated in at least one low-impact sports activity, with cycling (25%, 8/32), walking (19%, 6/32) and fitness/gymnastics (16%, 5/32) being the three most frequent types of performed sports (Tables 3, 4). Of the three patients who preoperatively participated in a sport at the competitive tournament level, two patients returned to their sports at the tournament level: the first at dancing (prior diagnosis TS = 10, at last follow-up TS = 8; Fig. 2a, b, Additional file 1: Video S1) and the second at playing soccer (prior diagnosis TS = 9, at last follow-up TS = 7). The third patient postoperatively switched sports from American football to cycling. The previously performed sports activities of basketball, badminton, judo, fishing and American football were no longer performed postoperatively by any patient (Table 4).

**Table 3:** Sports activities performed by each patient prior to diagnosis and at the time of last follow-up

Patient	Prior to diagnosis	At the last follow-up
1	Tennis, cycling, jogging	Tennis, cycling, jogging
2	Tennis, cycling, hiking, swimming, skiing	–
3	–	–
4	Tennis, bowling	Cycling, walking
5	–	–
6	Cycling, jogging	Cycling, walking
7	Fitness	Cycling, fitness
8	Cycling, Nordic walking, walking, aqua sport	Nordic walking, jogging, swimming, walking
9	American football, cycling, fitness	Cycling, walking
10	Soccer, badminton	Cycling, fitness
11	Diving	–
12	Nordic walking, gymnastics, swimming	Gymnastics, aqua gymnastics, swimming
13	–	–
14	Fishing	–
15	Tennis, skiing	Tennis, skiing, golf
16	Swimming	–
17	Cycling	–
18	Horse riding	Walking
19	Triathlon	Cycling, sailing
20	Soccer	Soccer
21	Soccer, canoeing, cycling	Cycling
22	Horse riding	–
23	Swimming, fitness	Swimming, fitness, hiking, diving
24	Canoeing, judo	Canoeing, jogging
25	Dancing	Dancing
26	–	Hiking
27	–	–
28	Dancing	Dancing
29	Fitness	Fitness
30	–	Cycling
31	Diving	Diving
32	Basketball, dancing	Dancing

**Table 4:** Sports activities performed prior to diagnosis and at the last follow-up, ranked by frequency, as given in brackets

Prior diagnosis	At the last follow-up
Cycling (7)	Cycling (9)
Gymnastics/fitness (5)	Walking (6)
Swimming (4)	Gymnastics/fitness (5)
Tennis (4)	Dancing (3)
Soccer (3)	Swimming (3)
Dancing (3)	Jogging (3)
Nordic walking (2)	Hiking (2)
Skiing (2)	Tennis (2)
Walking (2)	Diving (2)
Diving (2)	Nordic walking (1)
Canoeing (2)	Skiing (1)
Triathlon (1)	Aqua sport (1)
Jogging (1)	Canoeing (1)
Aqua sport (1)	Sailing (1)
*American football (1)*	Golf (1)
*Badminton (1)*	Soccer (1)
*Fishing (1)*	
*Bowling (1)*	
*Judo (1)*	
*Basketball (1)*	

Sports activities that are not performed anymore postoperatively are shown in italics

### Complications

A total of five complications as defined by Henderson et al. were observed in our cohort, with the most common reason for revision surgery being a periprosthetic infection (Henderson type 4, *n* = 3). Furthermore, there was one soft tissue failure (Henderson type 1) and one aseptic loosening (Henderson type 2) [24].

### Beneficial and limiting factors

Patients in whom preservation of the axillary nerve was possible had overall higher postoperative functional outcome scores (MSTS and TESS) and achieved higher WASs overall than patients in whom the axillary nerve had to be resected in order to achieve wide surgical margins (Table 5).

**Table 5:** Association of demographic and clinical factors with postoperative outcome scores

Factors	Yes	No	*p* value
Male	MSTS: 18 (IQR 12–24)	MSTS: 18 (IQR 11–24)	*p* = 0.270
	TESS: 83% (IQR 70–88)	TESS: 77% (IQR 64–83)	*p* = 0.596
	TS: 5 (IQR 4–6)	TS: 5 (IQR 4–6)	*p* = 0.850
	WAS: 6 (IQR 2–10)	WAS: 5 (IQR 0–13)	*p* = 0.650
BMI (< 30)	MSTS: 16 (IQR 12–20)	MSTS: 18 (IQR 12–24)	*p* = 0.734
	TESS: 79% (IQR 76–82)	TESS: 80% (IQR 67–86)	*p* = 0.907
	TS: 2 (IQR 0–4)	TS: 5 (IQR 4–6)	*p* = 0.081
	WAS: 3 (IQR 0–6)	WAS: 5 (IQR 2–10)	*p* = 0.532
Intraarticular tumour resection	MSTS: 19 (IQR 12–25)	MSTS: 17 (IQR 13–20)	*p* = 0.795
	TESS: 81% (IQR 59–87)	TESS: 78% (IQR 71–86)	*p* = 0.952
	TS: 5 (IQR 4–6)	TS: 5 (IQR 3–6)	*p* = 0.920
	WAS: 6 (IQR 2–14)	WAS: 2 (IQR 0–5)	*p* = 0.070
Preservation of the axillary nerve	MSTS: 21 (IQR 14–26)	MSTS: 14 (IQR 11–19)	***p* = 0.027**
	TESS: 84% (IQR 70–93)	TESS: 76% (IQR 47–82)	***p* = 0.049**
	TS: 5 (IQR 5–6)	TS: 4 (IQR 3–6)	*p* = 0.193
	WAS: 11 (IQR 5–18)	WAS: 2 (IQR 0–4)	***p* < 0.001**
Reverse shoulder reconstruction	MSTS: 21 (IQR 17–26)	MSTS: 15 (IQR 12–22)	*p* = 0.116
	TESS: 83% (IQR 78–88)	TESS: 76% (IQR 61–85)	*p* = 0.158
	TS: 5 (IQR 4–6)	TS: 5 (IQR 3–6)	*p* = 0.578
	WAS: 7 (IQR 5–12)	WAS: 3 (IQR 0–7)	***p* = 0.032**
Radiotherapy	MSTS: 18 (IQR 11–26)	MSTS: 15 (IQR 12–24)	*P* = 0.593
	TESS: 84% (IQR 69–93)	TESS: 79% (IQR 65–85)	*p* = 0.404
	TS: 6 (IQR 5–7)	TS: 5 (IQR 3–6)	*p* = 0.124
	WAS: 10 (IQR 4–17)	WAS: 4 (IQR 0–7)	*p* = 0.037
Chemotherapy	MSTS: 18 (IQR 15–24)	MSTS: 16 (IQR 12–24)	*p* = 0.464
	TESS: 83% (IQR 71–92)	TESS: 77% (IQR 62–83)	*p* = 0.168
	TS: 5 (IQR 4–6)	TS: 6 (IQR 4–6)	*p* = 0.985
	WAS: 6 (IQR 2–10)	WAS: 4 (IQR 0–11)	*p* = 0.464
Revision surgery	MSTS: 12 (IQR 11–22)	MSTS: 18 (IQR 13–24)	*p* = 0.389
	TESS: 76% (IQR 45–87)	TESS: 80% (IQR 70–87)	*p* = 0.479
	TS: 4 (IQR 1–6)	TS: 5 (IQR 4–6)	*p* = 0.241
	WAS: 2 (IQR 0–6)	WAS: 6 (IQR 2–11)	*p* = 0.166
High level of sports prior to diagnosis^a^	MSTS: 18 (IQR 12–21)	MSTS: 18 (IQR 12–26)	*p* = 0.722
	TESS: 81% (IQR 66–93)	TESS: 79% (IQR 68–84)	*p* = 0.512
	TS: 6 (IQR 3–6)	TS: 6 (IQR 4–6)	*p* = 0.722
	WAS: 24 (IQR 2–34)	WAS: 6 (IQR 0–10)	***p* = 0.023**

Statistically significant* p* values are shown in bold

*MSTS* Musculoskeletal Tumor Society Score, *TESS* Toronto Extremity Salvage Score, *TS* Tegner Activity Score, *WAS* weighted activity score

^a^Defined as a WAS prior to diagnosis of > 10

Regarding the postoperatively performed level of sports, patients with a reverse shoulder reconstruction as well as patients who performed sports at a high level prior to diagnosis (WAS > 10) achieved higher WASs than their respective counterparts (Table 5).

With the numbers available, gender, obesity (body mass index > 30), handedness (surgery on the dominant vs non-dominant extremity), type of tumour resection (intraarticular vs extraarticular), chemotherapy and revision surgery during follow-up were not associated with higher/lower functional outcomes or higher/lower level of sports activity performed postoperatively (Table 5).

Prior to diagnosis, a total of ten patients performed sports activities involving the upper extremity. Postoperatively, five of those patients returned to upper-extremity-demanding types of sports, and the remaining five patients switched to alternative sports activities with more focus on the lower extremity (Table 6).

**Table 6:** Demographics and clinical and surgical details of patients who performed sports activities involving the upper extremity prior to diagnosis, and the respective postoperatively performed sports activities and outcome scores

Patient	Demographic and clinical details		Surgical details			Sports activities involving the upper extremity		Outcome scores			
	Age and gender	Entity	Type of resection	Type of shoulder reconstruction	Preservation of the axillary nerve	Prior to diagnosis	After surgery	MSTS	TESS (%)	TS	WAS
1	74f	Chondrosarcoma	Intra	Reverse	Yes	Tennis	Tennis	21	82	6	28
2	53m	Chondrosarcoma	Intra	Reverse	Yes	Tennis	Tennis, golf	27	93	6	23
3	28m	Osteosarcoma	Extra	Anatomic	Yes	Canoeing	Canoeing	28	98	5	8
4	19f	Ewing sarcoma	Intra	Reverse	Yes	Dancing	Dancing	26	94	5	20
5	21f	Osteosarcoma	Intra	Reverse	Yes	Dancing	Dancing	18	80	5	5
6	73f	Chondrosarcoma	Extra	Anatomic	Yes	Tennis	None	12	59	2	0
7	72m	Chondrosarcoma	Intra	Reverse	Yes	Tennis	Cycling	12	63	1	8
8	59m	Ewing sarcoma	Intra	Reverse	No	American football	Cycling	11	82	5	6
9	58m	Ewing sarcoma	Intra	Reverse	Yes	Soccer, badminton	Cycling	17	85	3	11
10	33m	Osteosarcoma	Extra	Anatomic	No	Canoeing	Cycling	18	72	3	2

*m* male, *f* female, *intra* intraarticular, *extra* extraarticular, *MSTS* Musculoskeletal Tumor Society Score, *TESS* Toronto Extremity Salvage Score, *TS* Tegner Activity Score, *WAS* weighted activity score

## Discussion

The main findings of our study are: (1) good to excellent functional outcomes are possible following PHR after resection of a primary bone sarcoma; (2) postoperatively, patients generally perform sports activities with a low to medium physical demand, although individual patients are able to return to high-impact sports activities; and (3) preservation of the axillary nerve, a reverse shoulder reconstruction and higher levels of performed sports activities prior to diagnosis (WAS > 10) seem to be associated with a higher postoperatively achieved level of sports.

The good to excellent functional outcomes of our cohort are in line with the findings of previous authors [25–29]. For PHR with an allograft prosthesis composite, Abdeen et al. reported a mean MSTS of 26 (IQR 14–27) in a cohort of 36 patients with a median age of 23 years who underwent this procedure after resection of a primary bone sarcoma, failure of a reconstruction following a previous tumour resection, and resection of a local recurrence [27]. The longest follow-up for PHR was reported by Kumar et al., who reported a mean MSTS of 24 [standard deviation (SD) 3.8] and a mean TESS of 72% (SD 23.2) after a mean follow-up of 9 years (range 2–20 years) in 30 patients with a median age of 34 years who underwent PHR for resection of various bone tumours, including primary sarcomas, metastases and giant cell tumours [26]. Regarding the indication for PHR, Böhler et al. described a homogeneous cohort of 49 patients with a mean age of 18 years (IQR 17–21) who all underwent PHR following the resection of an osteosarcoma but subsequent reconstruction with different types of prostheses, reporting a mean MSTS of 24 (21–26) [28]. Similar to our findings, and despite the heterogeneity of the implants used, Böhler et al. found that preservation of the axillary nerve and the deltoid muscle were associated with better functional outcomes [28]. Houdek et al. described a cohort of 83 patients with a median age of 57 years (SD 18) who underwent PHR with endoprostheses (56 patients) or allograft-prosthesis composites (27 patients) following the intra-articular resection of a metastasis or a primary bone sarcoma and subsequent reconstruction with a reverse shoulder reconstruction (30 patients) or a hemiarthroplasty (53 patients) [29]. Houdek et al. found that patients with a reverse shoulder reconstruction had better functional results regarding the MSTS (22 vs 19) and improved range of motion compared to patients with a hemiarthroplasty [29]. However, with varying indications for PHR (such as resection for a primary bone sarcoma or for metastases or as a revision surgery following a previously failed endoprosthetic reconstruction in non-oncologic patients) and the different prosthesis systems used, the reported patient cohorts become heterogeneous, diluting the conclusion and relevance for specific groups of patients—such as patients undergoing PHR for resection of a primary bone sarcoma with a modular megaprosthesis.

Postoperatively, patients in our cohort mainly performed low- to medium-impact types of sports activities. Matching results were reported by Lang et al., who described a cohort of 18 patients undergoing PHR for bone sarcomas, with 14 patients performing low-impact sports activities and only 2 patients participating in medium- or high-impact sports activities after surgery [23]. Furthermore, Lang et al. also observed a postoperative switch from preoperatively performed sports activities that are demanding for the upper extremity to sports activities that are more demanding for the lower extremity [23]. However, contrary to the findings of Lang et al., we found that five of ten patients in our cohort continued to perform upper-extremity-demanding types of sports, partially compensating for functional limitations by using the non-affected upper extremity (Additional file 2: Video S2, Additional file 3: Video S3) [23].

In order to guide patients’ and surgeons’ expectations, it appears desirable to identify factors that might influence the postoperative functional outcome and the ability to do sports. In the present study, a preserved axillary nerve, a reverse shoulder reconstruction and a higher level of sports activity (WAS > 10) prior to diagnosis were associated with better results. These findings are in line with previous studies, who have also highlighted the functional importance of the deltoid muscle and the axillary nerve; however, in order to achieve wide margins, it can become necessary to resect these structures, which is paramount for the oncological outcome [30, 31]. Furthermore, reverse shoulder reconstruction with a proximal humerus megaprosthesis has previously been shown to be associated with good postoperative functional outcomes in non-oncologic as well as oncologic patient cohorts [29, 32]. While Lang et al. found no beneficial effect of a reverse shoulder reconstruction on the postoperatively performed level of sports in their cohort, Guven et al. described good to excellent functional outcomes and no endoprosthetic complications in ten patients following PHR with a reverse shoulder reconstruction after resection of a bone tumour [25, 32]. The latter finding is in line with our findings and appears plausible considering the improved range of motion described for reverse megaprosthetic PHR [33]. Regarding the level of sports performed prior to diagnosis, Hobusch et al. could not find a correlation with the achieved level of sports in 16 patients undergoing proximal femoral replacement for bone sarcomas [7]. As, for obvious reasons, upper and lower extremity megaprosthetic reconstructions are not comparable when it comes to a return to sport activities, we hypothesize that patients who return to a sports activity that is demanding for the upper extremity might benefit from previous proficiency when it comes to developing compensatory mechanisms or switching dexterity. However, as this study is the first to describe this aspect, future studies are needed to confirm or refute this association.

## Limitations

We acknowledge several limitations to our study. (1) The study follows a retrospective study design and includes only a small number of patients, which may not fully represent the risk factors. However, to the best of our knowledge, our study represents one of the largest and most homogeneous cohorts of patients who underwent PHR with a single-design megaprosthesis at a single institution, reflecting the rarity of these entities and this procedure. (2) Some patients could not be contacted and, despite the follow-up rate of 84%, it is possible that patients lost to follow-up may have poorer outcomes. Therefore, the results presented here may be considered high-end estimates. (3) Patients included in this study are considered to have been free of disease over the long term, so functional results and the performed level of sports might be biased, as patients who died during follow-up might have had lower functional outcome scores, participated in lower levels of sports, or even performed no sports activity at all. Again, this makes the results presented here likely to be a high-end estimate.

## Conclusion

A good to excellent functional outcome is possible after PHR following sarcoma resection. While low-impact activities appear to be more common, some individuals are able to participate in high-impact sports activities. Preservation of the axillary nerve, a reverse shoulder reconstruction and a higher level of sports prior to surgery were associated with better functional outcome scores and a higher level of postoperative sports activity.

## Supplementary Information

**Additional file 1: Video S1.** An initially 18-year-old female patient taking part in a professional dancing competition 5 years after PHR following resection of an osteosarcoma

**Additional file 2: Video S2.** An initially 53-year-old male patient playing golf 20 years after PHR following resection of an Ewing sarcoma

**Additional file 3: Video S3.** An initially 27-year-old male patient kayaking 10 years after PHR following resection of a chondrosarcoma

## Data Availability

The datasets used and analysed during the current study are available from the corresponding author on reasonable request.

## Abbreviations

IQR: Interquartile range
MSTS: Musculoskeletal Tumor Society Score
MUTARS: Modular universal tumour and revision system
PHR: Proximal humeral replacement
SD: Standard deviation
SSV: Subjective Shoulder Value
TESS: Toronto Extremity Salvage Score
TS: Tegner Activity Score
WAS: Weighted Activity Score
